# Low-Cost IoT-Based Sensor System: A Case Study on Harsh Environmental Monitoring

**DOI:** 10.3390/s21010214

**Published:** 2020-12-31

**Authors:** Ali Imam Sunny, Aobo Zhao, Li Li, Sambu Kanteh Sakiliba

**Affiliations:** 1 Nuclear AMRC, Advanced Manufacturing Park, Brunel Way, Rotherham S60 5WG, UK; ali.imam@namrc.co.uk (A.I.S.); aobo.zhao@namrc.co.uk (A.Z.); 2 Solartech-UK Ltd., 11 Llansannor Drive, Cardiff, Wales CF10 4BW, UK; info@solartechuk.com

**Keywords:** low-cost sensor, energy harvesting, wireless sensor network, IoT, harsh environment, condition monitoring

## Abstract

Wireless Sensor Networks (WSNs) are promising technologies for exploiting in harsh environments such as can be found in the nuclear industry. Nuclear storage facilities can be considered harsh environments in that, amongst other variables, they can be dark, congested, and have high gamma radiation levels, which preclude operator access. These conditions represent significant challenges to sensor reliability, data acquisition and communications, power supplies, and longevity. Installed monitoring of parameters such as temperature, pressure, radiation, humidity, and hydrogen content within a nuclear facility may offer significant advantages over current baseline measurement options. This paper explores Commercial Off-The-Shelf (COTS) components to comprise an installed Internet of Things (IoT)-based multipurpose monitoring system for a specific nuclear storage situation measuring hydrogen concentration and temperature. This work addresses two major challenges of developing an installed remote sensing monitor for a typical nuclear storage scenario to detect both hydrogen concentrations and temperature: (1) development of a compact, cost-effective, and robust multisensor system from COTS components, and (2) validation of the sensor system for detecting temperature and hydrogen gas release. The proof of concept system developed in this study not only demonstrates the cost reduction of regular monitoring but also enables intelligent data management through the IoT by using ThingSpeak in a harsh environment.

## 1. Introduction

According to the Nuclear Decommissioning Authority (NDA), on 1 April 2016 the total amount of radioactive waste is estimated to be 4.77 million m^3^ [1]. This increasing amount of waste material, which needs to be stored, treated, and disposed of in a proper manner, presents a great number of technical and temporal challenges for the nuclear industry [2]. In the decommissioning site at Cumbria, United Kingdom, the majority of the country’s nuclear wastes are currently stored on site. Depending on the type and radioactivity, varying storage strategies are applied within this site. One of the legacy storage facilities at Sellafield requires extraction of the historic Magnox Swarf, which is followed by packaging the material for interim storage before the final processing of the geological disposal. Over many decades of interim storage, a monitoring system needs to be implemented in order to predict the correct chemical evolution of the waste, which is mainly affected by the release of hydrogen gases and heat dissipation. This requires an assurance monitoring scheme in order to make sure that the hydrogen emissions and temperature of radioactive waste are well within the accepted parameter ranges.

The Internet of Things (IoT) is composed of numerous inter-related and interconnected devices, machines, and objects sharing data over a network aimed at reaching a common goal [3]. Being an enabling technology of Industrial Revolution 4.0, the goal of the IoT is to allow things and objects to be connected anytime and anywhere with anyone using any network, path, and service. Nowadays, the IoT is offering innovative solutions through advanced sensing technologies for major industries, e.g., healthcare services, the food supply chain, mining production, transportation and logistics, and firefighting [4]. The nuclear industry is no exception. One of the crucial aspects of the IoT is data management. Intelligent data management and data processing within the IoT system through the help of advanced Artificial Intelligence (AI) techniques [5,6] will further enhance the nuclear waste management productivity, safety and durability.

The primary parameters of interest in the condition monitoring of legacy nuclear waste containers are temperature variation and hydrogen gas release rate. Innovative ways to efficiently monitor these parameters from every container utilizing wireless data transfer could provide a greater level of safety assurance over a longer period of time. Numerous research projects have been carried out on Wireless Sensor Networks (WSNs) for varying applications, but the main difference with respect to the gaseous sensors used in WSNs is the power consumption. Some investigations are focusing on the implementation of a WSN-based air monitoring system. Each reported solution varies in the type of sensors tailored for the individual case for the purpose of optimizing power consumptions. To the best of the authors’ knowledge, there are no existing cost-effective devices that have been developed for the nuclear waste storage application. Different energy harvesting techniques have also been applied to develop such systems such as shown in Wang et al. [7]. They developed a wireless sensor network system where a solar panel is mounted on the sensor node, harvesting constant energy and providing adequate power to the sensors. A study on low-power Surface Acoustic Wave (SAW) sensors has been carried out in order to address the energy issues [8]. Furthermore, a reduction in power consumption has been adopted based on the duty cycle measurements using off-the-shelf sensors [9,10]. In addition, other low-power sensor systems have shown huge potential in harsh environmental conditions [11,12,13,14,15]. However, none of these sensors have taken into consideration the extreme environmental effects when deployed in real scenarios, such as high temperatures, release of hazardous gases, or changes in atmospheric conditions. Moreover, all of the aforementioned systems require bulky equipment to be connected in order to extract data.

The purpose of this paper is to develop a low-cost, compact, multimodal characterization sensor system with the current state-of-the-art, Commercial Off-The-Shelf (COTS) components. The IoT could provide an installed remote measurement system on each legacy nuclear waste container, thus providing a real-time understanding of the condition of the entire nuclear waste storage content deployed for in-situ or ex-situ measurements. In the paper, we have focused on the characterization of the sensors’ behaviors and firmware optimization. The outcome is a qualitative estimation of the gas concentration, which is essential to discriminate the small changes that will be presented as a result of changes in chemical reaction rates.

The paper is organized as follows: Section 2 describes the sensing principles, the sensor selection, and the proposed integration, followed by a description of the sensor calibration. Section 3 outlines the experimental setup. Section 4 presents the results and discussions. Finally, in Section 5, the conclusions and the future development perspectives are described.

## 2. Methods and Measurement Principles

This section presents the measurement principles and approach for implementing the robust sensing system. As low power consumption, along with cost effectiveness, is the main target, this section will delve more into how the COTS components have been selected in building a low-cost prototype sensor to demonstrate the concept for the nuclear waste storage application.

### 2.1. Sensing Principle and Circuitry

**Figure 1:**
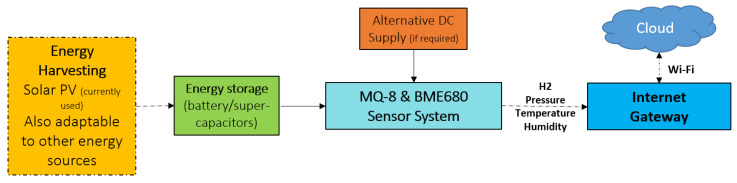
System block diagram for the sensing setup.

The sensor system is implemented by integrating multiple sensors with supplementary modules, such as energy harvesting, battery, charging circuit, and wireless communication for an Internet Gateway, to achieve real-time monitoring of the environmental parameters, e.g., H_2_, pressure, temperature, and humidity, as shown in Figure 1. The sensor system is constructed with harsh-environment-resistive sensors and provides accurate measurement for essential parameters in the environment. The energy harvesting module supplies the system with the necessary power, which extends the lifetime of the system and reduces the need for maintenance in long-term operation over decades. The wireless communication module provides a connection to the cloud server via the Internet gateway, enabling real-time monitoring and decision-making with the aid of Artificial Intelligence (AI).

**Figure 2:**
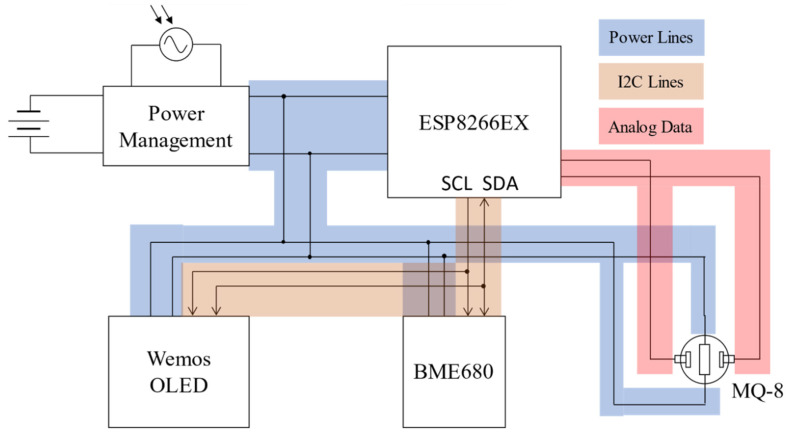
The sensor circuit wiring diagram.

The sensors used in the project are in the MQ family along with the Bosch Sensortec BME680. To achieve the sensing and monitoring function, the system is constructed using two solar panels for energy harvesting with a chargeable battery and a power management circuit, a hydrogen sensor MQ-8, a multifunction environment sensor BME680, a Wi-Fi Module ESP8266EX, and a Wemos organic light-emitting diode (OLED) display. The circuit connectivity can be seen in Figure 2. The power lines deliver the electricity from the energy harvesting and the power management module to the sensors and the communication module, which is shown as blue lines in Figure 2. The environment data are collected in two forms: analogue data and digital data. The digital data are transmitted via I^2^C buses, shown as brown lines. The Serial Clock Line (SCL) is sent by ESP8266EX as a clock signal and the Serial Data Line (SDA) as a bidirectional data signal. The analogue data are collected directly using the ADC (Analog to Digital) port of the microcontroller, which is shown as pink lines. Then, the ESP8266EX module can send the data to the base station via self-contained Wi-Fi protocol. The specifications of the components are introduced in the following section.

### 2.2. Sensor Integration

The power management is carried out by the solar panels and stored in the rechargeable battery for the operation of the entire system. The environmental data is collected by the Bosch Sensortec BME680, which is an environment sensor for gas, humidity, temperature, and pressure detection, and by MQ-8 sensors, and is transmitted to the local area network via Wi-Fi by ESP8266EX.

#### 2.2.1. Sensor Selection

Using a single sensor not only simplifies the system design but also reduces the energy consumption for environmental monitoring. However, there is no integrated sensor commercially available for sensing hydrogen gas together with other environmental parameters. Thus, the two sensors used in this system, namely MQ-8 and BME680, are suitable for monitoring the temperature and hydrogen release rate to prove the concept.

**Figure 3:**
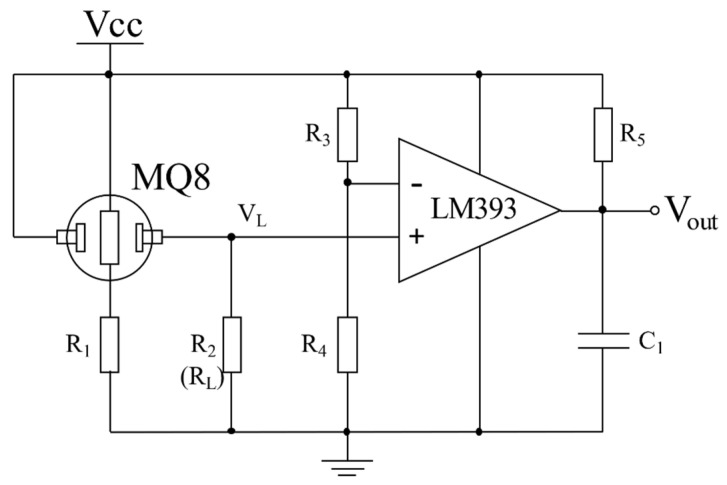
MQ-8 circuit diagram.

MQ-8 is a H_2_ detection sensor selected for its high sensitivity and is also able to detect a variety of other hydrogen-containing gases (e.g., nitrogen or air) [16]. The sensor uses stannic oxide (SnO_2_) as the sensing material, which has lower electrical conductivity within clean air. When the concentration of hydrogen gas increases, the conductivity of the material increases. Then, the variation in gas concentration within the environment can be converted from the conductivity change using a simple circuit, as shown in Figure 3. A heater voltage (V_H_) is introduced to supply a DC or an AC current to heat the sensor to working temperature. V_C_ is a DC power source for converting the conductivity change to a voltage signal with the presence of a load resistance R_L_, which is in series with the sensor. V_L_ is the voltage of load resistance R_L_ and can be connected to an amplifier circuit as shown in Figure 3.

BME680 is selected as a 4-in-1-sensor that can measure barometric pressure, relative humidity, ambient temperature, and gas concentration. The package is just 3.0 × 3.0 × 0.93 mm^3^, enabling a compact design. Another advantage of this sensor is its low temperature coefficient offset (TCO) [17]. The sensor provides digital data via I^2^C or a Serial Peripheral Interface (SPI) and operates with low current consumption (microamps) at a sampling rate of 1 Hz.

#### 2.2.2. Power Management

The sensor circuit can be operated at a very small current (approximately 5–20 mA) and 5 V DC, however, most of the current in this case is consumed by the heater circuit (around 45 mA). The used photovoltaics panel is able to deliver about 6.2 V and up to 300 mA, which is connected as an unregulated power source, through the battery to the ground and voltage input. Hence, a built-in regulator is employed to supply the sensor with constant 5 V. The DC–DC converter that is already embedded in the ESP8266EX Board converts the circuit voltage to 3.3 V. The power of the circuit (without the Hydrogen sensor) is 0.1 W. Therefore, the energy for one day is about 2.4 Wh/day, assuming that the power is on for 24 h. The MQ-8 sensor uses 0.5 W of power due to the embedded heater; hence, the energy for one day is 12 Wh/day. In short, the total power of the circuit including the hydrogen sensor MQ-8 is 0.6 W, and the total maximum energy consumption is 14.4 Wh/day.

#### 2.2.3. Communication Method and Display

There are several wireless communication protocols supporting wireless sensing and monitoring, e.g., Bluetooth and ZigBee. The advantage of Wi-Fi is that the sensor can be directly connected to a LAN and update the data to either a local control center or a remote one through the Internet. It is also supported by ThingSpeak^TM^ [18] to aggregate, visualize, and analyze live data streams in the cloud. The Wi-Fi Module based on an ESP8266EX chip offers a complete Wi-Fi networking solution and is also used as a controller for the sensors and the OLED display, which is the Wemos Mini D1 OLED. This module was used as a display module with the Wi-Fi Module based on the ESP8266EX, providing a user-friendly setup and debugging interface.

**Figure 4:**
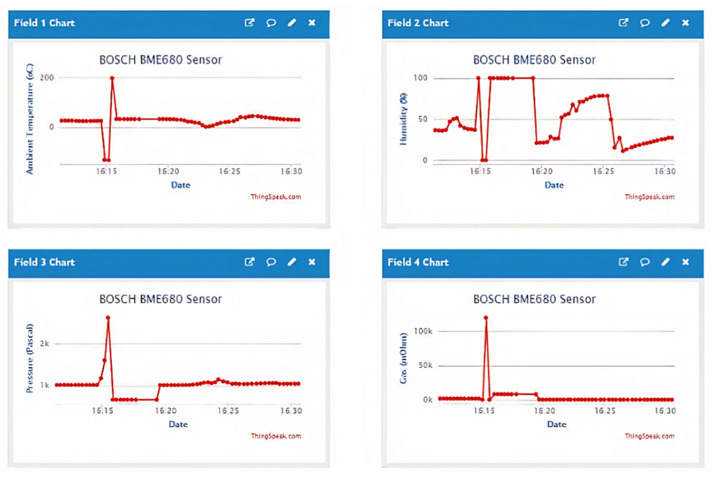
Example of data representation through the Application Programming Interface (API) provided by the open application platform Thingspeak.com.

A serial communication protocol with a baud rate of 115,200 is set and the device is connected to the Internet with a pre-set Wi-Fi Service Set Identifier (SSID) and password. Rather than implementing a classical PC-based user interface, our aim is to let the user access the data everywhere and, at the same time, increase the portability of the complete system. With a ThingSpeak private account and an Application Programming Interface (API) address, a Transmission Control Protocol connection is then established between the ESP8266EX and ThingSpeak cloud and the data can be monitored on the ThingSpeak API website, as shown in Figure 4. The sensor data shown are prior to calibration.

### 2.3. Sensor Calibration

The sensitivities of most sensors degrade over long-term operation when meeting the actual performance criterion. Hence, calibration is required to make the acquired sensor dataset more accurate. Generally, the result is ambiguous when the sample exerts more than one gas if it can sense both of the gases. To differentiate or to sense in a more efficient and better way, a new and advanced sensor is needed. The MQ-8 sensor is designed in such a way that it can recognize the number of gases simultaneously. The MQ family sensors are capable of measuring the concentration and substances that co-exist in a mixture. In the calibration process, we used a voltage sensor to adapt a voltage result of the gas sensor [19]. The first formula is used in [20], showing it is nonlinear for the sensor with gas concentration (1):

(1) $$R/R_{0}=\;{\left(1+k_{gas}C_{gas}\right)}^{-\beta }$$

where *R* is the sensor resistance, *R*_0_ is the sensor resistance, *C_gas_* the concentration of the used gas, *β* is the law of the characteristic power of the sensor, and *k_gas_* is the gas constant. The formula shows a power function with a negative exponent as:

*y* = m*x*^n^, n < 0 (2)

According to [20], the formula for measuring the clean air resistance with a known supply voltage $V_{CC}$ and a load resistance $R_{L}$ of 10 kΩ can be found as:

(3) $$R_{clean\_air}=\frac{R_{L}\left(V_{CC}-V_{out}\right)}{V_{out}}$$

where

$R_{L}\;$= Load resistance

$V_{CC\;}$= Sensor supply voltage

$V_{out}\;$= Output voltage

Therefore, the sensor resistance $R_{0}$ can be determined by the ratio of clean air resistance and air ratio as stated as below:

(4) $$R_{0}=\frac{R_{clean\_air}}{air_{ratio}}$$

*R_clean_air_* is the sensor reference resistance for clean air. *air_ratio_* = 9.56 and represents a constant of the MQ-8 sensor.

If *ppm* is the gas concentration in parts per million, then according to nonlinear regression, the output equation of the sensor is:

(5) $$ppm=m{\left(\frac{R_{gas}}{R_{0}}\right)}^{n}$$

(6) $$\log\left(ppm\right)=logm+nlog\left(\frac{R_{gas}}{R_{0}}\right)$$

(7) $$ppm=10^{logm+nlog\left(\frac{R_{gas}}{R_{0}}\right)}$$

**Figure 5:**
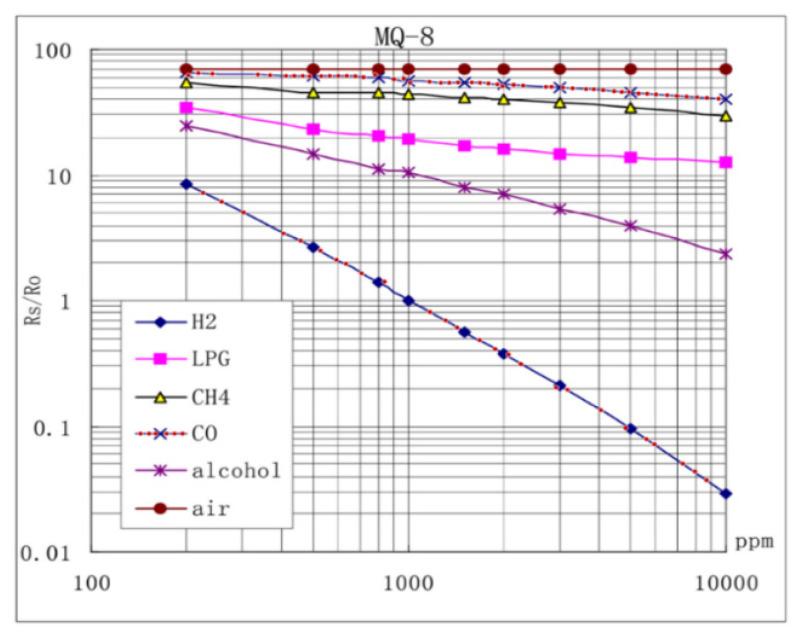
WebPlotDigitizer plotting for the MQ-8 sensor to extract the point for H_2._

In the equations, *R_gas_* is the sensor resistance in the presence of gas. The subscript *gas* stands for certain gases where *H*_2_ is hydrogen. From the datasheet, there is no formula provided for each gas type of MQ sensor. Using the datasheet’s graphical representations [16], we extracted the formula of gas and this is shown in Figure 5.

**Figure 6:**
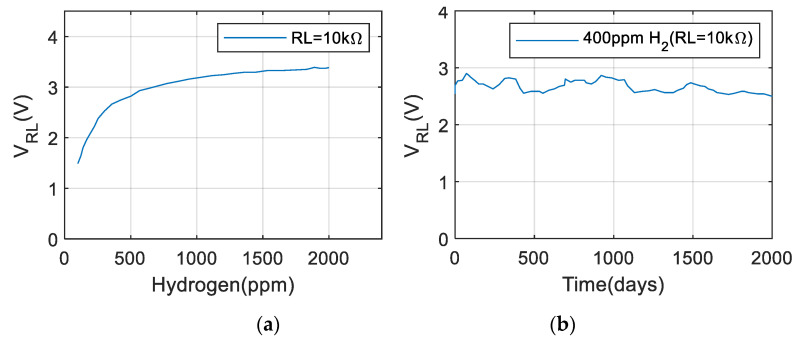
Sensor characteristics: (**a**) the sensitivity curve; (**b**) the long-term stability curve [16].

We used the MQ-8 sensor to extract the points on the graph ($log\left(ppm_{H_{2}}\right)$ = $logm+nlog\left(\frac{R_{H_{2}}}{R_{0}}\right))\;$ for H_2_. A set of points was extracted using WebPlotDigitizer to get a mathematical model that matches the data. Figure 6a shows the sensitivity curve, which shows the V_RL_ in hydrogen with different concentrations and that the resistance load R_L_ is 10 kΩ, and Figure 6b shows the long-term stability curve. The response graphs of the sensor provided by the manufacturer are plotted under standard conditions [16]. It provides the baseline of the characterizations for different practical applications, in this case, nuclear waste storage.

Similarly, the formulas, shown in Table 1, can be found experimentally by properly calibrating the MQ-8 sensor for a 1000 ppm H_2_ concentration in air and a value of R_L_ of about 10 KΩ (5 KΩ to 33 KΩ).

## 3. Experimental Setup

**Figure 7:**
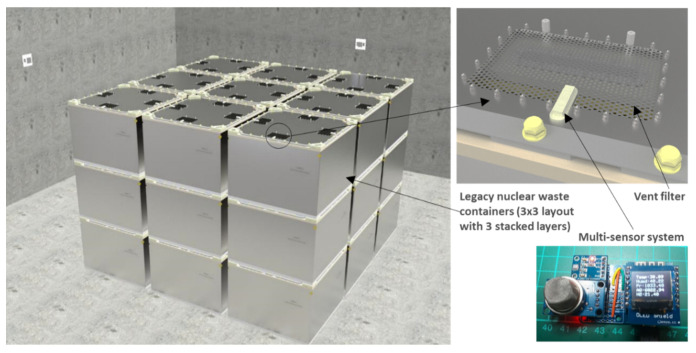
Concept design for legacy nuclear waste containers containing the four filters. The filters are recessed and screwed into the box lid, which provides two pathways to each filter.

The system is designed to perform under harsh environmental conditions and, in particular, the system is supposed to be deployed within the nuclear site. The sensors have been designed to be attached to legacy nuclear waste containers, which have an inner temperature between ~22 and 55 °C at normal room temperature. Additionally, the containers have a relatively low external contact radiation dose rate that does not cause any signal or data transmission interference [21]. However, the system at this stage has not been designed to be exposed to radiation, as it is in the conceptual design phase. Therefore, no shielding is required, and neither is a communication protocol with loss detection and re-transmission. A 3D model rendering image is shown in Figure 7, which depicts the stacking up of the legacy nuclear waste containers within the storage facilities. The containers have four filters on the lid to vent hydrogen gas to the ambient. The sensors can be fixed in the vicinity of the filters. Therefore, to replicate the scenario in a laboratory-based experiment, a small stainless steel cubic box was used for the validation of the multidetector sensor’s performance within the metal’s proximity.

Two scenarios are investigated:

- Scenario 1: Hot air flow test—pressure, gas, humidity, and temperature change inside and outside the stainless steel box under a hot air flow to monitor the temperature variation;
- Scenario 2: Hydrogen flow test—pressure, gas, humidity, and temperature change when a hydrogen flow is introduced into the stainless steel box to sense the hydrogen concentration.

**Figure 8:**
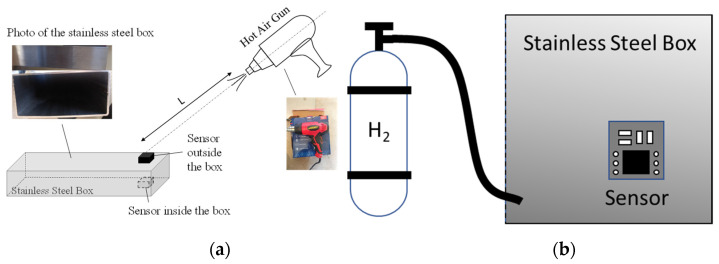
Experimental validation setup: (**a**) scenario 1 and (**b**) scenario 2.

In scenario 1, the temperature change is undertaken with a hot air flow passing through the surface of the sensor. Two conditions are tested individually: outside and inside the stainless steel box, and on the cap of the stainless steel bottle. The hot air flow is induced by a DEWORX Original 2000 W Hot Air Gun, which has two heating levels (600 °C and 300 °C) and two flow rate levels (300 L/min and 500 L/min). For both the inside and outside testing, the hot air gun is set to a fixed flow rate and held at a fixed distance above the sensor with the hot air flow directly pointing to the sensor. This can be seen in Figure 8a. 

In scenario 2, a hydrogen gas bottle is used to generate a hydrogen stream in the metal container and increase the hydrogen concentration level inside. The stream is produced at ambient temperature and guided into the metal box. This is shown in Figure 8b. The environmental parameters are monitored during the process for analyzing the influence of the hydrogen concentration increase.

**Figure 9:**
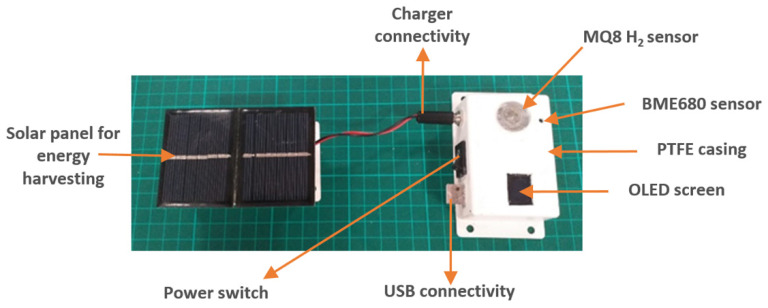
Compact robust multisensor system prototype in a thermally resistant PTFE case.

Figure 9 below shows the overall sensor system prototype. The total size of the prototype is only 3 cm high, 6 cm long, and 1.5 cm wide with the PTFE casing, which has a temperature tolerance of up to 250 °C, used to protect the electrical components.

## 4. Results and Discussion

This section evaluates the performance of the proposed system for analyzing the selected parameters under two different scenarios, which are explained below. The results obtained were processed in MATLAB and the varying characteristics of the sensor system are presented.

### 4.1. Scenario 1: Hot Air Flow Test

**Figure 10:**
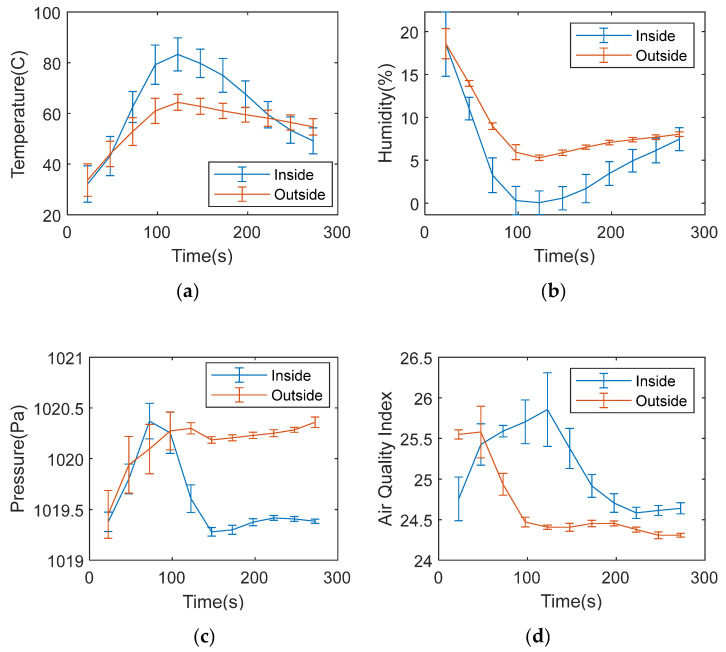
Scenario 1 test results for both inside and outside the stainless steel box: (**a**) Temperature; (**b**) Humidity; (**c**) Pressure; and (**d**) Air quality index.

The results were selected from 100 s before switching on the heater gun and then 280 s after, to give a steady reading. Ten individual tests were carried out and the results were averaged statistically. The divisions of different tests were calculated as error bars. To compare the trend of different parameters, these results were normalized by subtracting the average and then dividing by the standard division of the data set. The results are shown in Figure 10.

The results show that the temperature increases when the hot air gun switches on and starts to fall after the gun is switched off. The pressure and gas results show the same trend as the temperature change, as the pressure drops more quickly as the gun switches off, followed by the gas sensor results. It takes a longer time for the temperature to reset to a normal status due to the residual heat compared with pressure and gas. The humidity decreases during the increase in temperature and increases when the temperature goes down. This is because the humidity sensor measures the relative humidity of the surrounding environment. When the temperature increases, the saturate water vapor pressure increases, which decreases the relative humidity when the water vapor mass in the air remains the same.

The results also suggest that the recovery of the humidity is slower than that of the temperature. This is because the humidity sensor has a response time of 8 s, which means it takes 8 s to reach 63% of the total humidity change.

For the test results inside the stainless steel box, the hot air gun is switched on from 0 and switched off at 100 s. The trend in temperature, humidity, and pressure changes during the heating and cooling process are similar to the outside results, but in a slower path. This is reasonable because the interaction between the inner environments is either through the small window or via the heat interchange through the metal wall. Notably, the error bar between different tests is also smaller than the outside results for temperature and pressure measurement. It suggests that the inside measurement is more stable. 

### 4.2. Scenario 2: Hydrogen Flow Test

**Figure 11:**
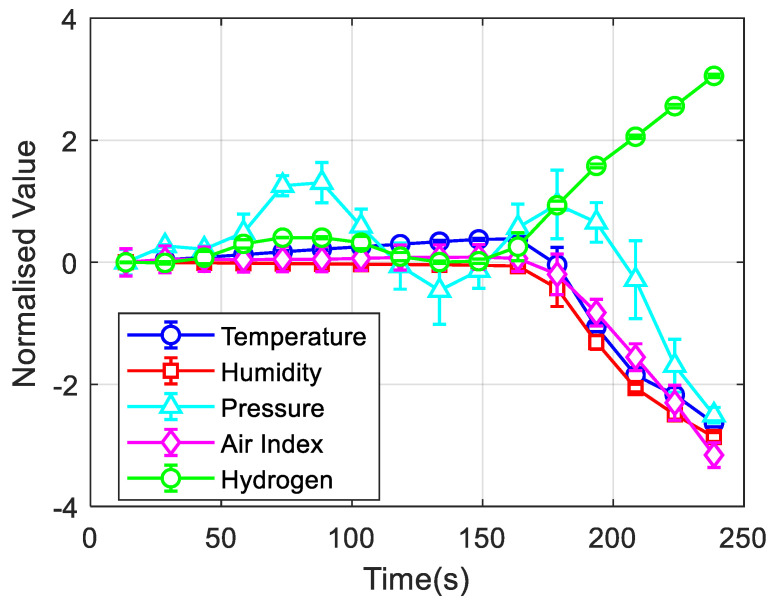
Scenario 2 test results for the detection of H_2_ components in comparison with the other environmental parameters.

This test shows the hydrogen detection characteristics in comparison with the other parameters. In the test, the hydrogen stream started to blow inside the stainless steel from 150 s and the parameter results were recorded to 240 s. The results for the hydrogen flow test are shown in Figure 11, which is used for comparing the parameter change during the increase in hydrogen concentration with other parameters. As the parameters are in different scales, they must be normalized to fit in one figure for comparison as shown in Equation (8) below:

(8) $$P_{norm}=\frac{P-\bar{P}}{\sigma }$$

where *P* stands for the parameter value, $\bar{P}$ is the mean of the parameter value in one test, and σ is the standard division of the parameter value in one test.

It is shown that the hydrogen concentration level starts to increase as the stream starts, which shows that the hydrogen sensor is working properly. At the same time, it shows a decrease in all other parameters. The decrease in temperature can be explained by the temperature decrease, which happens when the pressurized gas is released from the bottle. As there is no water content in the hydrogen stream, the humidity also decreased during the process. The pressure seems to decrease but is actually increasing and decreasing due to environmental influence.

## 5. Conclusions and Future Work

In this paper, we have presented a conceptual multisensor system that is able to monitor the environmental parameters for nuclear waste storage containers or other harsh environmental applications. The proposed system architecture breaks down the IoT structure into functional blocks and provides simple means of modularity between the layers. The approach is cost-effective, consumes less power, and is faster with respect to developing a new silicon sensor device. The research also describes the calibration methods for the MQ family of gas sensors for detecting leakage of H_2_ along with the radiation-tolerant environmental monitoring sensor BME680. The formulae for calibration of this type of sensor have been extracted from the data representation of the sensitivity curve. The sensor performs with higher sensitivity towards the desired parameter characterization in a preset environment. For both the experiments of temperature monitoring and H_2_ flow testing, the measured parameters from the proposed sensor demonstrate the expected results. The current system is self-powered by a solar cell from which the energy is stored in a researchable battery. Other energy-harvesting techniques for indoor storage facilities, e.g., from gamma radiation, ambient and transmitted radio frequency, conducted heat from nearby containers, or a resonant magnetic field, should be investigated in the future. In addition, the signal interference and electronics shielding in a radiation environment should also be studied.

The robust sensing system provides an alternative way for in-situ online monitoring of gaseous changes and future work will include the design of a radiation-shielded case for sensors that can be readily deployed in a harsh environment. The work will enable the installation and routine operation of a permanent monitoring solution for legacy nuclear waste containers or any other radioactive waste containers, in order to support the decommissioning strategy in the country. This paper has demonstrated the possibility of using the IoT technology for inventory management in nuclear industries.

## Figures and Tables

**Table 1:** Formula obtained for the MQ-8 sensors for measuring different gases [16].

Gas	$\log\left(ppm\right)=logm+nlog\left(\frac{R_{clean-air}}{R_{0}}\right)$
***H*_2_**	logm=−4.23 and n=3.09
***LPG***	logm=−2.53 and n=1.88
***CH*_4_**	logm=−2.55 and n=2.26
***CO***	logm=−7.36 and n=6.48
***Alcohol***	logm=−4.5 and n=4.82

## Data Availability

Data sharing not applicable.
